# A Perspective of the Diagnosis and Management of Congenital Tuberculosis

**DOI:** 10.1155/2016/8623825

**Published:** 2016-11-24

**Authors:** Manou Irmina Saramba, Dongchi Zhao

**Affiliations:** Department of Pediatrics, Zhongnan Hospital of Wuhan University, Wuhan, China

## Abstract

Tuberculosis continues to be a prevalent disease in the world and a global public health issue in many countries. The disease is more complicated in pregnant women because it imperils unborn offspring and results in congenital tuberculosis later if undiagnosed and untreated. Congenital tuberculosis is rare entity and an uncommon disease along with a high mortality rate. Congenital tuberculosis, a severe clinical type of tuberculosis caused by* Mycobacterium tuberculosis*, is a serious and fatal disease if left untreated. Our study emphasizes that it is necessary and mandatory to consider congenital tuberculosis in the differential diagnosis of neonatal or pulmonary infections in infants, essentially in countries where the incidence of tuberculosis is high burden. Mother to neonatal transmission of disease is well known via transplacental transmission through the umbilical vein to the fetus, through the ingestion of infected amniotic fluid. Early detection is challenging, because of the nonspecific nature of the signs and symptoms in tuberculosis during pregnancy and infancy. The degree of clinical suspicion is the essential component of diagnosis. Furthermore, it generally has a difficult treatment and it should not be delayed while waiting for diagnostic test results. Prompt identification and proper treatment regimens for congenital tuberculosis strongly relate with enhanced outcomes.

## 1. Introduction

Tuberculosis (TB) is one of the leading infectious diseases worldwide and is still a serious public health problem in many countries. World Health Organization (WHO) estimates that one-third of the population is infected with TB and the infection rate increases nearly 1% per year [1]. TB is relatively common in pregnant women; the prevalence of active TB in pregnant and postpartum women from high burden countries is upper to 60 cases per 100 000 population per year and from low burden TB countries, the prevalence is lower to 20 cases per 100 000 population per year or it is lower about 10 cases in total [2]. Although rare, vertical transmission carries a poor prognosis. The incidence of TB on pregnancy is increasing in countries with limited socioeconomic resources. In most of these countries, increased incidence is associated with increased prevalence of TB in the population of women of childbearing age. Latent pattern TB in pregnancy are correlated with a high risk of progression to active pattern that increases the risk of transmission from infected mother to child in the first 3 weeks of life. Congenital TB is caused by* Mycobacterium tuberculosis* acquired either in utero or at delivery. The disease is a rare complication in utero TB infection due to maternal haematogenous spread. Congenital TB is hard to diagnose because it is seldom discernable from other neonatal or congenital infections, thereby the case of congenital TB has been broadly underreported. In the second or third week after childbirth, the symptoms usually emerge and precocious diagnosis is essential. However, the clinical presentation of TB during pregnancy and infancy is often nonspecific, making recognition difficult [3]. Only 300 cases were reported in the scientific literature till 1989; subsequently, 58 cases in 1994 and, from 2001 to December 2005, 18 more cases have been mentioned [4]. In spite of difficult diagnosis, clinical suspicion and family history support diagnosis of congenital TB and precocious diagnosis avoids death in infancy period. The incidence of congenital TB is low; however, its significance lays on a mortality rate of up to 50% [5].

## 2. Objectives

This review aims to expose the identity of rare and fatal congenital tuberculosis if untreated and highlight the clinical features and difficulty of diagnosis and awareness among pediatricians to perform immediate therapeutic management from the beginning of suspicion. This study proposes to detect that the prognosis is poor for newborns, followed by a fatal evolution when diagnosis is delayed. This review also shows the variability and nonexistence of uniform consensus for disease treatment in neonatology.

## 3. Methods

An electronic search was carried out at PubMed, Embase, Cochrane library, and Google search engine to realize this review. Search was limited to literature and studies published in English, French, and Spanish language. The keywords used were neonatal, congenital tuberculosis, and antitubercular therapy, either separately or in different association. Data were collected from case reports in Africa, Asia, Latin America, Middle-East, United State, and Europe.

### 3.1. Definition

Tuberculosis in the neonatal period classically has been divided into two types: congenital and postnatally acquired. Congenital tuberculosis was first described by Beitzke in 1935 based on autopsy review. Essential diagnostic element included isolation of* Mycobacterium tuberculosis* from the infant and either a primary hepatic complex or other discoveries consistent with TB disease in the first days after birth (excluding postnatal exposure) [6]. Considering the mortality, the criteria for the introduction of isoniazid in 1952 became less pertinent as the number of autopsies decreased.

In 1994, Cantwell et al. suggested revised criteria. The main criterion continues to be referred throughout the first week of life in newborn infants by the identification of* Mycobacterium tuberculosis* infectious lesions. Considering a more modern approach to diagnosis; however, the secondary criteria were expanded. Less-invasive technics have been established to replace widely the open abdominal surgery that is very heavy for the newborn. However, liver biopsy remains an acceptable alternative to a primary liver complex in order to identify caseating granulomas. Discovery of* Mycobacterium tuberculosis* in the female genital tract who has just given birth or in placenta was also added to the secondary diagnostic criteria for congenital disease. Exclusion of postnatal acquisition by thorough investigation closely of the contacts remains an important secondary criterion [7]. Diagnosis of congenital TB can be established by the primary criteria and at least one of the secondary criteria.

### 3.2. Mother to Infant Transmission of TB

Pregnancy creates a state of physiological immunosuppression, and TB in pregnant women is relatively common [8]. The placenta plays a main role for a highly effective natural barrier preventing the spread of bacteria toward fetus, the vertical transmission of TB in children is obviously uncommon, and the documented cases of congenital TB are noticeable by their scarcity. On the other hand, the postpartum contamination happens far more often. Congenital TB has been presumed as an infection acquired in utero, owing to the age of the infant, lack of any known contact with an active case of TB, and generalized spreading of the disease [4].

The mode of acquisition for congenital infection is assumed to be via maternal bacillemia transmitted to the placenta, amniotic fluid, and/or maternal genital tract. Before penetrating to the infant's liver or lungs to form a primary tuberculous complex, bacilli cross the placenta through the umbilical vein, and a primary focus develops in the fetal liver with involvement of the periportal lymph nodes and the bacilli infect secondarily the lung. Therefore it is a hematogenous infection, and hence it is called congenital infection by vertical contamination of TB [9–11]. Otherwise, the neonate may acquire the disease in utero or in intrapartum and/or at childbirth through aspiration or inhalation or ingestion of infected amniotic fluid causing primary infection of fetal lungs and gut or via direct contact with the infected maternal genital tract. Mostly, the bacilli arriving in the fetal lungs remain inactive during the fetal period and until after birth. The existence of substantial increase in oxygenation and pulmonary circulation may activate TB after birth. Dissemination is carried out via fetal circulation to other organ systems and may engender events such as TB meningitis [12]. Therefore, transplacental infection arises late in pregnancy and aspiration from infectious amniotic fluid occurs in the perinatal phase. Precocious postnatal contamination from a family member or close contact is also possible. Regarding breastfeeding and TB, breast milk is intact of contamination so transmission of disease does not occur [11, 13].

### 3.3. Clinical Presentations of Congenital TB

Clinical presentation of TB in the neonatal period is identical if the acquisition of the disease is congenital or postnatal. Furthermore, known effects of TB in pregnancy are marked with the following problems: infertility, poor reproductive performance, repeated abortions, stillbirths, preterm labour, and premature rupture of membranes. As regards fetus, it may have intrauterine growth retardation and low birth weight and has increased risk of mortality. The average age of manifestation of congenital TB is 24 days (range, 1 to 84 days) [7].

Infected infant is sometimes usually born prematurely, but symptoms of illness can arise from birth to several days or weeks of age and may occur acutely or chronically. Signs in neonatal tuberculosis are frequently nonspecific or unusual presentations and their mothers are seldom symptomatic. All of these lead to a difficult diagnosis. There are no clinical focuses which are pathognomonic for congenital TB presentations. The most common presentation is with poor feeding, fever, irritability, failure to thrive, failure to gain weight, cough, respiratory distress, hepatosplenomegaly, splenomegaly, lymphadenopathy, and abdominal distension [14].

More severe presentations are meningitis, septicaemia, miliary, unresolving, or repeated pneumonia, and disseminated intravascular coagulation. Lethargy, jaundice, ascitis, otitis media with or without mastoiditis, parotitis, osteomyelitis, paravertebral abscess, cold abscess, and papular or pustular skin lesions are different known features. On the other hand, obstructive jaundice owing to glands in the porta hepatis may also arise; some of cases may have progressive deterioration of liver function in absence of respiratory symptoms [15].

Less common manifestations such as apnea, facial palsy, vomiting, cyanosis, jaundice, seizures, and petechiae have been reported in less than 10 per cent of cases. Hemophagocytic syndrome can occasionally arise in rare instances. In addition, lung and liver are more frequently involved in congenital TB. They are the target organs [13]. Lastly, the course is frequently fulminant, considered in many cases by dissemination of the infection [16].

### 3.4. Diagnosis and Investigations

The most important element of the diagnosis is a high index of clinical suspicion. Congenital TB is particularly challenge to diagnose. It is not straightforward to differentiate between congenital and early postnatally acquired TB. Tuberculosis in the neonate more often has no specific signs or unusual presentations and their mothers seem seldom healthy, and in this manner the diagnosis of TB is paramount. Although congenital TB is a comparatively rare pattern or subtype, subsequently diagnosis remains difficult and may be missed. So detailed history of maternal health or infection such as notion of latent TB infection (LTBI) and of family contact has to be given particular consideration in the first line to support the early diagnosis of congenital TB thus preventing death in infancy period.

During pregnancy the immune changes and it creates a state of physiological immunosuppression. Thereby, in pregnant women, performing the screening of a potential latent* Mycobacterium tuberculosis* infection in order to prevent maternal-child TB disease is very important. Pregnancy is a way to screen for LTBI, but to identify pregnant women carrying latent infection remains a challenge, because pregnancy eliminates the T-helper 1 (Th1) proinflammatory reaction, which may dissimulate symptoms while rising susceptibility to novel infection and reactivation of TB. There are two current screening tests for diagnosing LTBI. The tuberculin skin test (TST) has low cost and requires no laboratory infrastructure but has low specificity and a potential cross-reactivity with BCG and environmental mycobacteria and it requires the patient to return in 48–72 hours. In contrast a new second blood test, interferon-gamma release assay (IGRA), is more specific and suppresses the need for a return visit, with no cross-reactivity with BCG or ecological bacteria, but has high cost and needs laboratory infrastructure lacking in many hospitals in developing countries [2, 17].

Thresholds interpretation of the TST or IGRA does not change during pregnancy; a positive TST is induration ≥10 mm for HIV-negative and ≥5 mm for HIV-positive pregnant women whereas a positive IGRA is a difference in IFN-*γ* concentration of >0.35 IU/mL QuantiFERON Gold Test In-Tube (QGIT) or >6 spots (T-spot.TB) between the TB antigen and negative control sample, regardless of HIV status. In low incidence settings an IGRA may therefore be more specific and less sensitive than TST in pregnancy. On the other hand, in a high burden setting, IGRA may be more sensitive than TST; so in pregnant women from high burden setting, some studies show that a positive IGRA is associated with an increased risk of developing active TB [2, 18]. There is a long-held suspicion that pregnancy-induced immune suppression affects LTBI screening tests, but studies on the relative findings of the tests in pregnancy occur exclusively from low incidence settings countries and show divergent findings. Contrariwise, no studies had shown the comparison of performance of both tests in pregnant women in a high incidence setting countries. The Centers for Disease Control and Prevention (CDC) states that IGRAs can be used in place of TST and are preferred in BCG-vaccinated individuals and those unlikely to return for interpretation. WHO does not recommend routine use of IGRAs for latent TB screening. WHO recommends latest for high burden countries of TB to continue using the TST for LTBI screening, because it performs similarly to IGRAs and has low cost. In several studies, LTBI faced the challenge of having no gold standard for diagnosis [17]. However, further studies and methods are required for diagnosis of LTBI in pregnant women.

Obstetrician and trained nurses should be assets to inquire antenatal history and it is very beneficial in early diagnosis and resolving newborn outcome. However, it is helpful to investigate maternal genital tract, placenta's morphology, and histology in suspected cases at the time of delivery. Screening of household or family contacts may yield source of information about infection. In neonates, there are no exact signs and symptoms of congenital TB. Later these signs and symptoms are attributed to other causes like prematurity, congenital viral infections or bacterial sepsis, and acute or chronic intrauterine infections [19, 20]. As the signs and symptoms may mimic bacterial infection and the condition does not improve with broad-spectrum antibiotics or conventional treatment, it should alert and suspect the pediatrician for diagnosis in an ill newborn infant.

The noninvasive and moderately efficient methods could be performed in newborns in order to have samples for microscopy and culture including gastric aspirates, sputum (induced), tracheal aspirates (if mechanically ventilated), skin lesions, ear discharge, ascitic fluid, cerebrospinal fluid (CSF), and pleural fluid (if present) for acid fast bacilli and cultured on standard egg based media for 12 weeks [13]. In contrast, bronchoalveolar lavage (BAL) is an imperative examination, because discovery of* Mycobacterium tuberculosis* DNA in BAL fluid by polymerase chain reaction (PCR) is an efficient method, for diagnosis in newborn [21]. Liver or lymph node biopsy may be undertaken for histology and culture but it remains highly invasive method for neonate. Moreover, early morning gastric aspirates to infants with pulmonary illnesses have a 75% positive yield, which is remarkably higher than older children [9]. Standard workup is less efficient in investigation for TB for neonate, such as complete blood count, C-reactive protein, erythrocyte sedimentation rate, liver function tests, and chest radiograph which may also be imperative and informative.

In addition, a liver ultrasound may also be imperative and if abnormal features are discovered or the diagnosis is in doubt, a liver biopsy should be carried out to estimate for caseating granuloma formation. Although, in the neonatal period, meningitis is a scarce presentation, a lumbar puncture with culture for acid fast bacilli (AFB) is recommended. A fine needle aspiration of lymph node may also be requisite to detect AFB in case of superficial adenopathy.

In many cases of congenital TB illustrated, over 95% of cases related in mother were discovered as having open TB [22]. However, at the time of diagnosis of the newborn, most of mothers are unmindful of their infection [9]. Accordingly, if the disease is suspected in the mother then screening and investigation of the mother for TB are paramount. Indeed, if the mother is identified to be suffering from active TB, it will be important to revise her genital tract and the placenta in order to evaluate the presence of AFB and granulomata. While the test is not often positive in 10% to 15% of cases and may only get positive several months after infection, tuberculin skin test (TST) in newborn is recommended in the investigation of congenital infection. This could be explained in neonates and infants less than 6 weeks. Since the immaturity of the immune system makes random TST, there are more recent investigations which have not been validated in children younger than the age of 5 years, as realizing assays that rely on interferon-gamma release, habitually, produce unspecified results [11, 23]. Finally, once the mother and newborn are labeled TB, both should undergo for Human Immunodeficiency Virus (HIV) testing because increased TB is often present in HIV infected patients.

### 3.5. Management of Congenital TB

When a woman with TB gives birth, the purpose is to warrant TB-free survival of her neonate. In the general point of view, congenital TB is scarce; thereby no therapeutic trials were conducted to improve the treatment of optimal manner. As a result, there is no tangible guideline to manage congenital TB. The treatment is the same in case of postpartum acquired TB. Congenital TB is always fatal if untreated. However, treatment should begin as soon as the diagnosis of active tubercular lesion is suspected without waiting for laboratory confirmation of results. Appropriate culture specimens should be obtained fast.

For mother and newborn, drug susceptibility in both is crucial, particularly given the existence of multidrug-resistant tuberculosis (MDR TB). However, several therapeutic regimens have been assessed and established. In that case, newborns suffering from pulmonary and extrapulmonary disease should undergo a regimen of four drugs like older children and adults with active disease until sensitiveness is known [4, 9].

Sometimes the discovery of TB in the mother is related to the suspicion of the disease in the newborn, occasionally the opposite. However, if the mother is suffering from the disease, after having been labeled with the different investigation, the implementation of treatment is paramount. The therapeutic regimen is the same. There are two types of treatment of tuberculosis, namely, first-line treatment using isoniazid, rifampicin, pyrazinamide, and ethambutol; second-line treatment involving aminoglycosides, capreomycin, ethionamide, fluoroquinolones, cycloserine, and para-aminosalicylic acid [24].

Treatment regimens should include at least 2 and preferably 3 drugs to which the organisms are presumably susceptible which are isoniazid, rifampin, pyrazinamide, and an aminoglycoside, such as amikacin or streptomycin. Nowadays the accepted method of treatment is isoniazid (10–15 mg/kg/day), rifampin (10–20 mg/kg/day) and pyrazinamide (15–30 mg/kg/day), and either streptomycin or ethambutol (15–25 mg/kg/day). For first 2 months, both drugs isoniazid and rifampicin should be streamlined to the patient's treatment after 2 months which are given for an additional period of 4 to 10 months. In the instances of more severe presentation such as tuberculosis meningitis and endobronchial and miliary disease, corticosteroids are recommended. Supportive therapy such as oxygen may be nevertheless required [4, 9]. An essential element of treatment is the close observance of regularity and duration of treatment. The infant should be closely monitored on a monthly basis to clinical point of view for adverse side effects to antitubercular therapies. Habitual physician-patient contact is deeply desirable. Regarding visitors and hospital staff, it is necessary to avoid for any transmission of active disease, and indeed family member visits and hospital staff should undertake precautions by avoiding transmission between neonates.

## 4. Discussion

Tuberculosis remains a major public health scourge. As congenital TB continues as a rare presentation of a frequent infectious disease worldwide, only 300 cases have been reported until 1989. Since then, more than 80 additional new cases have been reported and documented. Most of the study that have been collected and analyzed consists of individual case reports, cases series, and reviews. Congenital TB is estimated at 2% in countries with high TB endemic. It is lower in developed countries, making it an unknown disease and difficult to evoke [10]. The mortality rate is very important and high, nearly 50%, which is often due to delayed diagnosis followed to delayed treatment [3], and 22% among patients receiving chemotherapy [7]. Furthermore, newborn infants who suffer from congenital TB after 4 weeks of age have a 77% existence rate compared with 44% survival in those who suffer before 4 weeks [25].

According to the criteria suggested by Beitzke in 1935 and reviewed in 1994 to Cantwell, the diagnosis of congenital TB necessitates the existence of TB lesions reported in newborns. The demarcation or distinction between the acquired neonatal patterns of congenital type and neonatal acquired postnatal is still challenging to determine in clinical practice and has only epidemiological interest. Our study has observed that most of case reports used the criteria of Cantwell for the diagnosis of congenital TB.

There are two essential components of the Koch bacilli paths for congenital TB. Approximately, it has been noted that 50% of cases meet each of these paths [11]. Primarily in utero, it can be spread from the mother to the fetus through transplacental transmission, so the organisms reach the fetus through the umbilical vein, thus creating a primary focus of* Mycobacterium tuberculosis* complex in the liver of neonate with secondary hematogenous spread. Secondarily, by direct infected amniotic fluid yearning or ingestion induces formation of a primary* Mycobacterium tuberculosis* complex in the gastrointestinal tract or the lungs. It appears if the caseous lesion in the placenta ruptures immediately into the amniotic cavity, and another explanation at delivery via inhalation of infected amniotic fluid through direct contact with the infected maternal genital tract. This entire route is described as true congenital TB.

The second main route of infection seemed more probable in five case reports studies that we explored in those studies reporting that the patient had no liver damage departing, proven by the absence of liver enlargement with result of normal liver function. But respiratory and intestinal symptoms were present. The chest radiographic results showed increased pulmonary marking and bilateral pulmonary infiltrates. Abdominal radiographic has shown a nonspecific intestinal dilatation [5, 25–28].

In the opposite direction of those studies described above, the first transmission route was assumed in three case studies, with abdominal distention, progressive liver dysfunction, and absence or presence of simple respiratory symptoms with chest X-ray incidence normal [8, 15, 29]. Some case reports also showed the two transmission route types. This situation was observed in the case study conducted by Bor et al. [30], Dewan et al. [31], and Bonnet et al. [32].

Congenital TB is a rare entity of TB. Except for diagnostic challenge and underreporting, the scarcity of the state is yielded by the fact that genital TB or tuberculous endometritis in childbearing women usually leads to infertility. This readily explains one of the reasons for low incidence of congenital TB. Those women may still be able to procreate, due to advances in current assisted reproduction technology, as were the cases presented by Wong et al. [26] and Flibotte et al. [33]. Moreover, this circumstance is very risky because it still generates very complicated congenital TB. Therefore, it is always necessary to take a complete history of the patient prior to in vitro fertilization (IVF) [26, 33–36].

The more severe type of TB such as extrapulmonary, miliary, and meningeal TB in mother presents high index risk factors for congenital TB in newborn infant and threatens future offspring. Vertical transmission does not occur from mothers with tubercular pleural effusion or generalized adenopathy. In contrast, Peng et al. [22] noted in their analysis that 22 mothers with pleural TB were able to pass on the infection to her child. The vertical transmission rate is between 0% and 16%. Vertical transmission appears less among mothers with pulmonary TB and to patients who already initiated treatment before childbirth. However, it is more common in patients with miliary and genital patterns of TB [11]. In addition, scientific information regarding the increased risk of congenital TB to mothers with resistant TB or concurrent HIV infection in literature is less. Mothers who have finished antitubercular treatment (ATT) before delivery or given ATT for at least two weeks duration before delivery are less probable to spread the disease to the newborn infant as compared to untreated mothers [13].

In most case reports we have studied, it is noted that to discern the signs of congenital TB remains a challenge for all practitioners. So, prompt diagnosis is difficult, because of mimicry signs to other infections [4]. So to make the correct diagnosis is almost impossible during one day after birth. Classically, infected newborns are born prematurely with low birth weight for gestational age. As said in literature, symptoms appear only in newborns after third week of life, with a median age of 28 days [5]. On the other hand, there have been cases of late onset up to 3 months of life or over, as the case study by Vogel et al. has shown [34], describing the appearance of congenital TB manifestation at 154 days after birth. And another study reported the case on day 112 [37]. The clinical features comprise hepatosplenomegaly with liver and spleen lesion, abdominal distension, lymphadenopathy, and ascites; pneumonia with respiratory distress, military, nodular, or lymphadenopathy in imaging finding, and no improvement in spite of use of broad-spectrum antibiotic treatment; meningitis with involvement of cranial pairs of facial nerve, lymphocytosis, and decrease of biological levels of glucose and protein in cerebrospinal fluid; and sepsis and finally other banal signs [11, 22, 38, 39]. But none of them is pathognomonic of congenital TB. Several authors demonstrated that the complication of delay diagnostic of congenital TB incorporates military, meningitis TB, and otitis media, resulting in seizures, deafness, and death [40]. The last complication is common, proven in most cases we have seen [33, 41, 42].

However, it is of utmost importance to document the antenatal history of mother owing to lack of pathognomonic sign following diagnostic difficulty. Thereby, up to 60–70% of mothers have no symptoms and their babies also [5]. The knowledge of a potential latent or active* Mycobacterium* TB infection during pregnancy is crucial. As in the case report study by Aelami et al. [43], Doare et al. [44], Tomar et al. [35], and Chemsi et al. [10], the screening of mother is an essential component. A recent study by Peng et al. establishes that 162 mothers had open TB throughout pregnancy or postpartum period. Of them, 121 had no past history of TB before getting pregnant and were diagnosed through pregnancy [22]. In a review of 32 cases of congenital TB, 24 of the mothers were asymptomatic [8].

Furthermore, Singh et al. [45] described another opportunity to make the diagnosis of congenital TB. They reported clinical and laboratory findings for TB investigation. It includes newborn infant mainly from endemic areas unresponsive to conventional treatment for worsening pneumonia, if the mother was labeled to have TB and baby has no exact symptoms, when the cerebrospinal fluid was discovered to have a high lymphocyte count in the nonexistence of any identifiable bacterial pathogen and in manifestation of fever and hepatic and splenic enlargement. Other diagnostic opportunity has been elucidated in several case reports we have seen. Despite having features suggestive of congenital TB, infants are often categorized as septicemia, and the real diagnosis would be delayed. The likelihood of perinatal TB should be considered even in newborn infants suffering unresponsive pneumonia [31].

Investigation includes TST, early morning gastric aspirate for acid fast bacilli, and* Mycobacterium* culture in all body fluid and biopsy swab from lymph node and liver. The histology and culture of placental biopsy sample should be performed and evaluated. The TST is generally negative in newborn infants at first manifestation, while a study conducted by Bor et al. [30] suggests that TST reaction was positive (16 mm). Frequently, the TST converts to a positive result months later after the first negativity result. Correspondingly in another study of 9 infants with congenital TB, only 2 presented the positive reactions (>10 mm) [4]. Tuberculin skin testing is positive in less than 15% of cases of congenital TB while gastric or tracheal aspirates are positive for TB in 80% of cases. A recent report proved the value of bronchoalveolar lavage as a technique of isolating the mycobacteria in perinatal TB [21]. Liver biopsy is really possible because it can have a very high diagnosis sensibility, which is 100% [25].

Recently, interferon-gamma (IFN-*γ*) release assays have not been authenticated in children younger than the age of 5 years. They may be accepted in combination with TST but should not be utilized as a substitution [9]. In contrast, Zheng et al. [46] attested T-spot assay in their case, based on IFN-*γ* release assays (IGRAs) from specific T cells in vitro in response to stimulation of* Mycobacterium tuberculosis* antigens and which has objectified a positive result in a premature newborn, conceived by in vitro fertilization.

Sometimes radiographic finding in some of cases is uneventful at the onset, but if diagnosis and treatment are delayed, radiographic progression will be poor very quickly. In our study, we noticed that discovery of miliary TB on chest X-ray is frequent in several index cases [36, 40, 47]. In 143 cases of congenital TB, chest radiographical findings reviewed by Peng et al. [22] which are 46.8% represented a miliary pattern. Also, given the case reports we have analyzed, hepatosplenomegaly dominates the imaging abdominal finding associated with multiple focal lesions, as were the cases presented by Raj and Sarin [28], Lee et al. [48], and the analysis study carried out by Peng et al. [22] (44,7% of 143 cases).

Finally, to complete the investigation, endometrial biopsy and histological examination of placenta are useful. Several studies followed this method, but sometimes mother declines to carry out it.

No precise treatment regimens for management of congenital TB are recommended. Treatment contains isoniazid, rifampicin, ethambutol, and kanamycin or amikacin for the first two months followed by isoniazid and rifampicin for 6–12 months or similar to miliary tuberculosis or isoniazid, rifampicin, and pyrazinamide along with streptomycin and kanamycin for 9 to 12 months [13]. Most specialists also advise using corticosteroids for tuberculosis meningitis to reduce both mortality rates and neurologic sequelae. The use of corticosteroids in other severe patterns of congenital TB infection, such as endobronchial and miliary disease, can also be accepted [9]. Note that most experts use the treatment scheme for 9 to 12 months as long as there is low immunologic ability in infants [49]. Timely identification and proper treatment regimens for congenital TB highly correlated with upgraded outcomes. According to the institution criteria by Cantwell et al. in 1994, mortality rates from published cases were about 45% [9]. Several experts evoke and emphasize the improvement in mortality rate after the implementation of previously elucidated criterion. It is mandatory to investigate the antenatal history of the mother to best use these criteria and immediately institute the treatment under high suspicion. The risk of untreated tuberculous disease in newborn infant is higher than the side effects with antitubercular therapies. So it is always beneficial to treat the condition rather than leaving it untreated.

For some TB pregnant women, their diagnosis is essential as it will cause a fatal situation for the offspring, if not labeled. The treatment is the same as in nonpregnant women but has to be careful with side effects, especially when it is a multiple resistance drug TB. Streptomycin should be used with caution in pregnant women because it can harm fetus. The admission of another drug pyrazinamide also is risky because its teratogenic effect is unknown and is not mentioned [12].

Prevention should be possible through early and timely detection of disease throughout pregnancy; institution of proper therapy especially in endemic area and TB screening should be included in prenatal investigation care [4, 41]. So, the knowledge of a potential latent* Mycobacterium* TB infection is key to avoid maternal-child active TB. In low burden countries, the CDC recommends latent TB screening only for high-risk women but pregnancy is not considered high risk by itself. In high burden countries, latent TB screening is not routinely recommended [2]. The women with LTBI reactivated throughout pregnancy has a high risk of death, antenatal complications, and poor fetal outcomes. In those with a positive LTBI test, isoniazid (INH) preventive therapy (IPT) can restrict the risk of developing active TB by as much as 60%. The CDC currently recommend 6–9 months of IPT for pregnant women with a positive LTBI test, including those from high burden setting, noting that no studies have directly explored the safety of INH in pregnancy. In contrast, INH may increase the risk of hepatotoxicity in pregnancy, though a decision-analysis study concluded that proper monitoring minimizes the risk. It crosses the placenta but does not cause significant fetal toxicity [17]. In most of the studies, WHO does not recommend routine practice of IGRAs for latent TB screening in contrast to CDC. However, more research has to be done on LTBI in pregnant women.

## 5. Conclusion

The diagnosis of perinatal TB requires a high index of suspicion and thorough evaluation of both mother and infant is mandatory to establish the diagnosis. In the absence of an associated history of maternal TB, it is difficult to diagnose clinically the disease whereas it is easy to commence timely treatment. This review highlights that when maternal TB is not treated, a delayed diagnosis in children leads to a fatal prognosis and death can occur after an overwhelming sepsis. The need to develop specific protocols to deal with this situation is mandatory. Finally, in poor response to conventional antibiotics therapy in newborn especially in endemic areas for tuberculosis or if the mother has risk factors for tuberculosis, practitioners should be conscientious to evoke a congenital tuberculosis.
